# Evaluation of interventions led by pharmacists in antimicrobial stewardship programs in low- and middle-income countries: a systematic literature review

**DOI:** 10.1017/ash.2024.342

**Published:** 2024-11-11

**Authors:** Tatiana Aporta Marins, Graciele Riveres de Jesus, Marisa Holubar, Jorge L. Salinas, Gabrielli Pare Guglielmi, Vivian Lin, Silvana Maria de Almeida

**Affiliations:** 1 Hospital Israelita Albert Einstein, São Paulo, SP, Brazil; 2 Stanford University, Stanford, CA, USA

## Abstract

**Objective:**

We performed a systematic literature review to identify and describe pharmacist-led antimicrobial stewardship programs (ASPs) interventions in low- and middle-income countries (LMICs).

**Design:**

Systematic literature review.

**Methods:**

We searched PubMed for studies evaluating pharmacist-led ASP interventions in LMICs from January 1, 2012, to November 4, 2023. We evaluated the article’s country of origin, described ASP interventions, and analyzed their reported outcomes.

**Results:**

Twenty-four studies were included; ten were conducted in China, two in India, two in Thailand, five in Africa, three in Latin America, and two in the Middle East. The predominant interventions in the studies were education and training followed by audit and feedback. The outcomes reported included reduction in antimicrobial consumption, cost reduction, shortening of the duration of antimicrobial therapy, and de-escalation.

**Conclusions:**

Our findings reinforce the importance of clinical pharmacists leading interventions related to antimicrobial stewardship in LMCIs and the global importance of investing in Infectious Disease training.

## Background

Antimicrobial resistance (AMR) is a global public health problem associated with approximately 5 million deaths in 2019.^1^ Studies cite that countries with lower social development status often have higher mortality rates related to AMR.^1,2^ Unfortunately, the COVID-19 pandemic has negatively impacted the efforts to control AMR in hospitals.^3–6^ Actions to halt the emergence of AMR, including the prevention of infection and the proper use of antimicrobials, should be reinforced in all healthcare settings.^7–10^

Since the first global AMR report by the World Health Organization was released in 2014, several health agencies have released strategies that include the implementation of antimicrobial stewardship programs (ASPs). ASPs are included as an important strategy within health institutions to promote the adequate use of antimicrobials, ultimately, slowing the emergence of AMR.^11^ Developing countries risk AMR for its high consumption of antimicrobials, moreover, another major obstacle are their lack of structured ASPs.^12–16^ Furthermore, the operation mode of ASPs often varies due to differences in available resources, workforce, technology, local medical culture, and insufficient support from hospital administration.^17^

The CDC, Centers for Disease Control and Prevention, suggests that pharmacists are the ideal professional to lead implementation efforts to improve the use of antibiotics. Studies have shown the importance of infectious diseases (IDs) pharmacists in the success of the ASPs.^18–20^ However, the small number of certified ID specialists limit their engagement in these programs. Clinical pharmacists could play a key role in managing the use of antimicrobials, as they are directly involved in dispensing and monitoring these drugs. However, in reality, in many countries, especially in low- and middle-income countries (LMICs), their role in antimicrobial management is not as developed.^12,21^ Given this scenario, the aim of our review is to identify and describe pharmacist’s interventions in LMICs on ASPs.

## Methods

### Systematic literature review and inclusion and exclusion criteria

This review was conducted according to the Preferred Reporting Items for Systematic Reviews and Meta-Analysis (PRISMA) statement.^22^ The steps to choose articles relevant for this review were: identification of the theme, development of the guiding research question, definition of inclusion and exclusion criteria, and choice of databases for the search. Then, we evaluated the abstracts, selected them according to their eligibility and contributions to the topic, interpreted and compiled the results, and synthesized the data that were described in the discussion stage. The guiding research questions were: “What are the interventions led by pharmacists in antimicrobial stewardship programs in LMICs?” The established inclusion criteria were studies performed in inpatient settings and studies that focused on the role of pharmacists in hospitals located in LMICs with emphasis on pharmacist-led interventions on ASP. To classify countries in LMICs, we used the World Bank classification as a reference.^23^ Letters to the editor, commentaries, conference abstracts, and review articles were excluded.

### Search strategy

The literature searches were performed in Medical Literature Analysis and Retrieval System Online (MEDLINE/PubMed). Considering that in the last decade, different health agencies have published on the importance of implementing ASPs, this review covers publications from January 1, 2012, to November 4, 2023, using the following keywords: “antimicrobial stewardship program or antibiotic stewardship,” “pharmacist or pharmacist-led or pharmacist-driven or clinical pharmacist,” “low- and middle-income countries or developing countries.” The terms were combined with each other through the Boolean operators “AND” and/or “OR.” We also reviewed the reference lists of retrieved articles to identify studies that were not identified from the preliminary literature searches.

### Data abstraction and quality assessment

Titles and abstracts of all articles were screened to assess whether they met inclusion criteria. Abstract screening was performed by one reviewer (TAM). Two reviewers (TAM and GRJ) independently abstracted data from each article which included: country, population, study design, study period (months), primary objectives, interventions carried out by pharmacists, evaluated antimicrobials (dosage, indication, duration of treatment, de-escalation, therapeutic duplicity, adherence to guidelines), and final outcomes, such as AMR, costs, and length of stay. Reviewers settled disagreements by consensus. We assessed the risk of bias using the Downs and Black scale.^24^ Quality analysis was performed independently, and discrepancies were resolved after consensus among reviewers.

## Results

**Figure 1.**
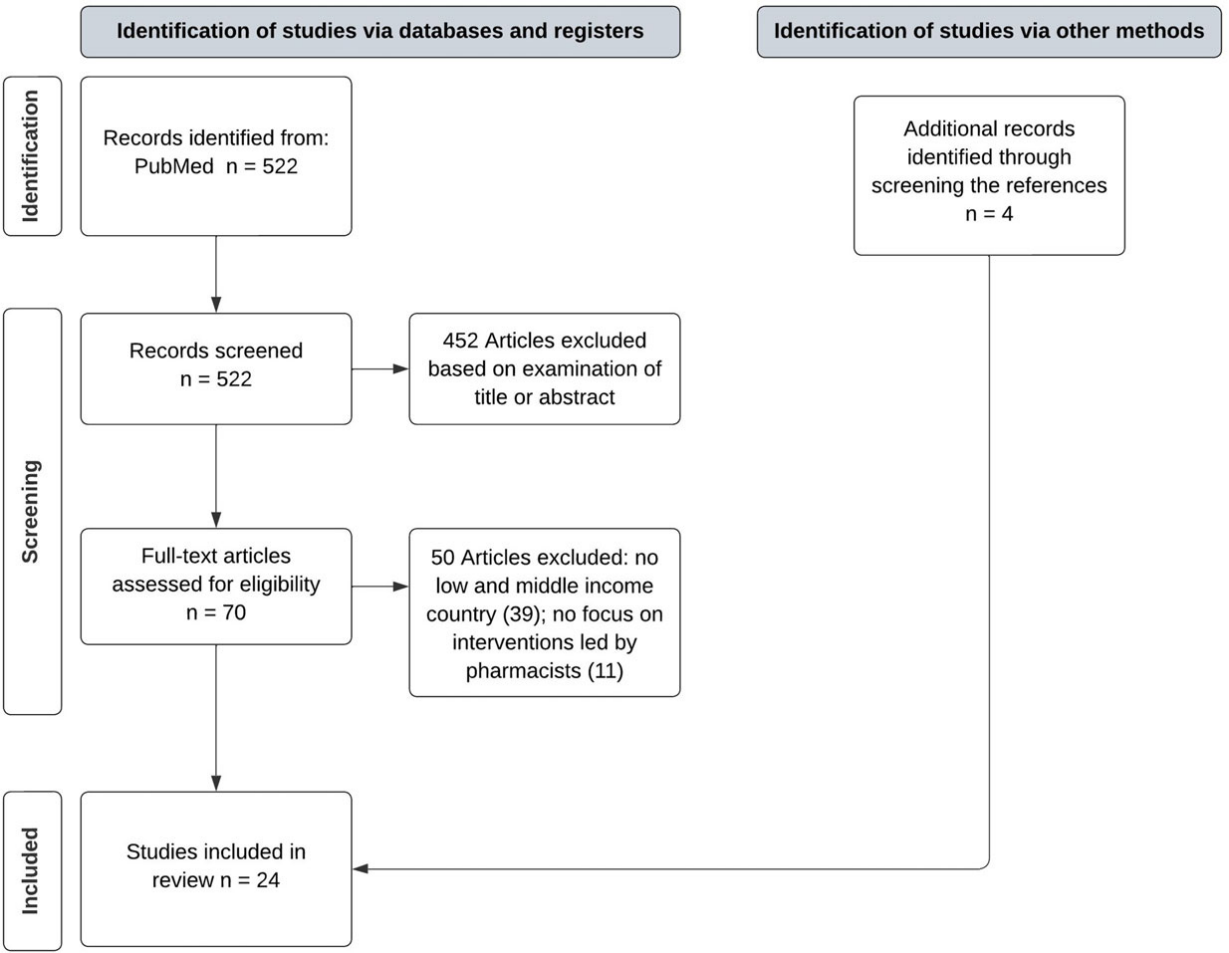
PRISMA flow chart showing the review selection process.

In total, 522 citations published between January 2012 and October 2023 were identified in the database search. After screening by title and abstracts, 452 articles were excluded. Of the 70 articles remaining for full reading, 50 articles were excluded, and 4 additional studies were added by screening the references of the articles. In sum, a total of 24 articles met the inclusion criteria (Figure 1).

### Characteristics of included studies

We included twenty-four studies in the final review^25–48^ (Table 1). All studies were non-randomized; most of them were quasi-experimental studies^25,26,29–31,33,34,36,38,40,43–45,48^, five were prospective cohort studies ^27,32,37,46,47^, three were retrospective observational studies^28,39,42^, and two were pre-post intervention.^35,41^

**Table 1.** Summary of studies on Antimicrobial Stewardship Programs (ASPs) with pharmacist-led or pharmacist-driven classified by country or region

First author, year, location	Study design, study period (month) and dates, characteristic of included patients	Objectives	Antimicrobials studied	Outcomes	D&B Score	PharmDs as ID	PharmDs as nonspecialist
Apisarnthanarak, 2015, Thailand^25^	Quasi-experimental, 08 [January–September 2012]. Medical wards	To evaluate the evolution of the ASP program with hospital-trained ID pharmacists.	Not specified	Reduction in antibiotic consumption and duration of therapy and reduction in hospital stay after interventions	18		
Abubakar, 2019, Nigeria^26^	Quasi-experimental study, 7 [May 2016–December 2016]. Department of obstetrics and gynecology.	To evaluate the impact of pharmaceutical interventions on prescription, clinical and economic outcomes in two hospitals.	Ampicillin-cloxacillin, metronidazole and amoxicillin-clavulanic acid.	Increased adherence to protocol-recommended prophylaxis duration, decreased redundant antimicrobials, significant decrease in antibiotic utilization (DDD/procedure), decrease in mean cost of antibiotic prophylaxis	16		
Bergh, 2020, South Africa^27^	Prospective cohort study, 12 [July 2017–July 2018]. General ward and ICU/High care.	To evaluate the use of nonspecialist pharmacists to implement antimicrobial stewardship interventions for community acquired pneumonia (CAP).	Not specified	Decreased in length of stay (LOS) and infection related LOS in public hospital. There was no statistical difference in mortality.	19		
Cheng, 2023, China^28^	Retrospective cohort, 26 [January 2021–March 2023]. Neurosurgical ward	To evaluate the impact of pharmaceutical interventions for the treatment of central nervous system infections.	Vancomycin, meropenem, linezolid, ceftriaxone, polymyxin B and miscellaneous.	Reduction in antibiotic use density (eg, linezolid) and a significantly higher score for the rational use of antibiotics (p < 0.05).	15		
Díaz-Madriz, 2020, Costa Rica^29^	Quasi-experimental study, 48 [April 2013–March 2017]. Inpatients general.	To evaluate the impact of the pharmacy-led antimicrobial management program.	Meropenem, ertapenem, moxifloxacin, levofloxacin, ciprofloxacin, cefuroxime, ceftriaxone, ceftazidime, cefazolin, cefalotin, vancomycin, ampicillin/sulbactam, and linezolid.	Increase in the consumption of cefazolin in surgical prophylaxis and a decrease in the incorrect use of ceftriaxone and levofloxacin.	18		
Du, 2020, China^30^	Quasi-experimental study, 36 [January 2016–December 2018]. Gastroenterology ward.	Demonstrate the effectiveness of ASP through interventions led by a pharmacist in the gastroenterology department.	Not specified	There was no significant difference in DDD/100 patient-days antimicrobial consumption before and after the interventions. Regarding the proportion of patients receiving a combination of antibiotics, there was a significant reduction (p = 0.03) and significant reduction in the average length of stay (p = 0.00).	14		
Gebretekle, 2020, Ethiopia^31^	Quasi-experimental study, 15 [November 2017–January 2019]. Medicine and pediatric wards.	Verify the impact and viability of a pharmacist-led intervention on antibiotic consumption.	Vancomycin, meropenem, third-generation and fourth-generation cephalosporins.	Reduction in the duration of treatment and in DOT/1000 patient-days. Reduction in LOS and in mortality.	15		
Holguín, 2020, Colombia^32^	Prospective observational study, 13 [August 2016–September 2017]. Department not specified	Describe pharmaceutical interventions aimed at the consumption of broad-spectrum antimicrobials.	Piperacillin/tazobactam, ceftriaxone, ceftazidime, cefepime, ceftaroline, meropenem, doripenem, imipenem/cilastatin, ertapenem, ciprofloxacin, vancomycin, polymyxin B, colistin, linezolid, daptomycin, or tigecycline.	Pharmaceutical interventions were adhered to by 82.5% of the infectologists and de-escalation being the most frequent.	19		
Holguín, 2021, Colombia^33^	Quasi-experimental study, 13 [August 2016–September 2017]. Department not specified.	Demonstrate the impact of antimicrobial stewardship programs on antimicrobial consumption, duration of therapy and costs in a private clinic.	Piperacillin/tazobactam, ceftriaxone, ceftazidime, cefepime, ceftaroline, meropenem, doripenem, imipenem/cilastatin, ertapenem, ciprofloxacin, vancomycin, polymyxin B, colistin, linezolid, daptomycin, or tigecycline.	Decrease in consumption per DDD/100 patient-days and in costs associated with antibiotic therapy.	16		
Jantarathaneewat, 2022, Thailand^34^	Quasi-experimental, 08 [August 2019–April 2020]. Medical wards	To evaluate impact of ASP by comparing groups targeted by ID pharmacists compared to those who were not.	Third- and fourth-generation cephalosporins, β-lactam β-lactamase inhibitors, carbapenems, fluoroquinolones, glycopeptides, polymyxin, tetracyclines, phosphonic acid derivatives, and antifungals.	Greater adherence to ASP in the group led by the ID pharmacist in relation to indication, dosage, and adequacy.	17		
Kerr, 2021, Ghana, Tanzania, Uganda, Zambia^35^	Pre-post intervention*, 14 [February 2019–April 2020]. Department not specified.	Capacity building of pharmacists in Africa with support from the United Kingdom to implement protocols in the management of antimicrobials.	Not specified	In each country there was improvement in prescription development guidelines and antimicrobial management. Engaging the team with regular feedback and launching the CwPAMS Antimicrobial Guideline app increased adherence.	13		
Kuruvilla, 2023, India^36^	Prospective interventional study, 12 [July 2019–July 2020]. Surgical units	To analyze and determine the factors that contribute to the inappropriate use of antimicrobials through pharmaceutical interventions.	Glycopetide, tetracyclines, aminoglycosides, polymyxins, oxazolidindiones, carbapenems, quinolones, lincosamides, penicillins, nitromidazoles e cephalosporins	Significant improvement after pharmaceutical interventions in the appropriate use of antibiotics, including a decrease in treatment duration.	13		
Li, 2017, East China^37^	Multicenter prospective cohort, 4 [March–April 2014 and June–July 2014]. Hospitalization in ICUs	To assess the impact of pharmaceutical interventions on the use of antimicrobials.	Tigecycline, carbapenem, cephalosporins, quinolones, anti-MRSA and antifungal.	Decrease in the rate of bacterial resistance, reduction in the antimicrobial spectrum and shorter duration of empirical therapy.	16		
Mahmoudi, 2020, Iran^38^	Quasi-experimental study, 25 [April 2016–May 2018]. Emergency, internal medicine, surgery, cardiology, obstetrics/gynecology, and dermatology wards.	To assess the effect of implementing an antimicrobial stewardship program on the consumption of antibiotics.	Caspofungin, amphotericin B, voriconazole, colistin, linezolid, vancomycin, meropenem, and imipenem.	Reduction in the consumption of antimicrobials measured by DDD/1000 patient-days and a significant drop in the monthly cost, with the greatest savings for voriconazole. There was a decrease in length of stay, while changes in mortality was not significant.	14		
Masri, 2022, Lebanon^39^	Retrospective observational study, 12 [January 2019–January 2020]. Pediatric and adult patients hospitalized.	Evaluate the interventions of pharmacists before and after the implementation of a protocol for the use of carbapenems.	Meropenem, ertapenem, or imipenem/cilastatin.	The number of interventions increased and a significant increase in dose adjustment and antibiotic de-escalation according to DDD/100 bed-days.	17		
Messina, 2015, South Africa^40^	Quasi-experimental study, 13 [July 2013–August 2014]. ICU and general wards.	Evaluate adherence after implementing a national protocol conducted by pharmacists, administering antimicrobials within one hour of prescription.	Intravenous antibiotics unspecified.	Increased adherence to administering antimicrobials within one hour was demonstrated in the 33 hospitals included in the study by reviewing pharmacy dispensing processes and changing the profile of fixed administration hours.	15		
Nampoothiri, 2021, South India^41^	Pre-post intervention*, 36 [February 2016–January 2019]. Department not specified.	Describe the evolution of an ASP led by a pharmacist at the university hospital in south India and the challenges faced.	Colistin, amphotericin, caspofungin, ertapenem, linezolid, meropenem, anidulafungin e tigecycline.	After the third year of implementation, there was a reduction in the consumption of antimicrobials by DDD/1000 patient-days due to the increase in the adequacy of prescriptions through the main initiative to evaluate the duration of therapy.	12		
Wang, 2019, China^42^	Retrospective observational study, 77 [July 2010–December 2016]. Outpatients and inpatients.	Evaluate the impact of ASP on antimicrobial use and its correlation with antimicrobial resistance (AMR).	Carbapenems (imipenem/ cilastatin and meropenem), fluoroquinolones (levofloxacin, moxifloxacin and ciprofloxacin).	Decreased consumption of antimicrobials DDD/100 bed-days. Decreased the resistance rates of *E. coli* and *P. aeruginosa* to fluoroquinolones and decreased incidence rate of methicillin-resistant *Staphylococcus aureus* (MRSA).	14		
Wang, 2022, China^43^	Quasi-experimental study, 20 [March 2018–October 2019]. Two hepatobiliary surgery wards and two respiratory wards.	Impact of pharmacist-led antimicrobial stewardship on antibiotics consumption, costs and adequacy of use.	Broad-spectrum antibiotics.	Decrease in costs and in DDDs per patients in the two nursing specialties due to the reduction in antimicrobial consumption and the duration of empirical treatment.	18		
Xu, 2022, China^44^	Quasi-experimental study, 16 [March 2018–October 2019]. Department of vascular and interventional radiology.	Analysis of the rational use of antibiotics in the antimicrobial management program led by clinical pharmacists.	Thirty-one antibiotics	Decline in the utilization rate DDDs per patient-day and cost of antibiotics There were no significant differences in the length of stay between the two groups.	19		
Yu, 2023, China^45^	Quasi-experimental study, 11 [October 2021–September 2022]. Neurosurgical intensive care units (ICU)	Evaluate the impact of implementing the pharmacist-led AMS program.	Ceftazidime, piperacillin/tazobactam, cefoperazone/sulbactam, cefepime, imipenem-cilastatin, meropenem. Polymyxin, and tigecycline.	Reduction of use of broad-spectrum antibiotics and decrease the rates of multidrug-resistant bacteria	14		
Zhang, 2019, China^46^	Prospective cohort study, 8 [April 2017–December 2017]. ICU and ward departments	To analyze the effectiveness of clinical pharmacists’ consultation (CPC)	Not specified	Significant improvement in patient outcome after adherence to the clinical pharmacist’s suggestion.	20	40%	60%
Zhang, 2020, China^47^	Prospective and multicenter cohort study, 32 [April 2017–December 2019]. Inpatients with confirmed diagnosis of infectious diseases.	To analyze the effectiveness of Pharmacist-led Clinical Consultation (CPC) in the treatment of infectious diseases.	Not specified	Increased effective response in patients included in the study and improved prognosis after interventions in patients with controlled confounders.	21		
Zhou, 2021, China^48^	Quasi-experimental study, 10 [June 2018–March 2019]. Orthopedics department.	To analyze pharmaceutical interventions in antibiotic prophylaxis after skin test standardization.	Cephalosporin, vancomycin, clindamycin, and levofloxacin.	Significant decrease in the number of unnecessary skin tests and a reduction in the average cost of antimicrobials.	18		

Note. D&B, Downs and Black; DDD, defined daily dose; DOT, days of therapy; CPC, clinical pharmacist consultation; AMR, antimicrobial resistance; MRSA, methicillin-resistant Staphylococcus aureus; *E. coli*, *Escherichia coli*; ASP, antimicrobial stewardship program; ICU, intensive care unit; CAP, community acquired pneumonia; CwPAMS, Commonwealth Partnerships for Antimicrobial Stewardship; LOS, length of stay; ID pharmacists, pharmacists specialists in infectious diseases; nonspecialist, pharmacists nonspecialists in infectious diseases.

*Assessment by the authors as the study design was not specified in the reference.

**Figure 2.**
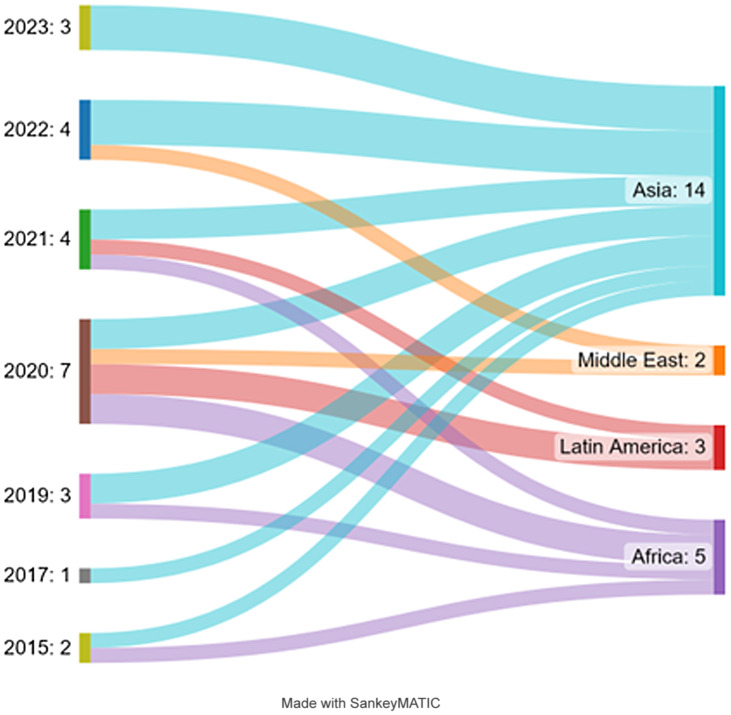
Distribution of articles by year of publication and geographic region Sankey Diagram.

Nearly half of the studies included in our review were conducted in China (10 studies)^28,30,37,42–48^, two in India^36,41^, and two in Thailand^25,34^. Five studies were performed in Africa^26,27,31,35,40^, three studies in Latin America^29,32,33^, and two in the Middle East^38,39^. Most of the studies were performed between 2019 and 2022^26,27,29–35,38,39,41–44,46–48^, three in 2023^28,36,45^, two in 2015^25,40^ and one in 2017^37^ (Figure 2).

The study duration varied from four to seventy-seven months. In sixteen studies, the pharmacists implementing the interventions were not specialized in ID.^26–31,35–40,44,45,47,48^ Six studies described ID pharmacists implementing the interventions^25,32,33,41–43^, while two studies had both nonspecialists and ID pharmacists.^34,46^ All included studies assessed patients admitted to tertiary care hospitals. Ten studies were conducted in medical and surgical wards, five in ward units and intensive care units, two in intensive care units, and seven did not specify the department. Most studies evaluated the consumption of broad-spectrum antimicrobials^26,28,29,31–34,36–39,41–45,48^ with exception of seven studies^25,27,30,35,40,46,47^ which did not specify the antimicrobials involved. The predominant interventions in the studies were education and training, mainly of medical teams and clinical pharmacists (11 studies)^26,29,30,35,39–44,48^, audit and feedback (11 studies)^26,27,29–31,38–41,43,45^, development of protocols addressing common infections like community acquired pneumonia, targeted de-escalation of carbapenems and treatment guides (10 studies)^25–27,29,32,35,38,39,41,47^, and daily rounds (6 studies)^25,28,30,36,37,44,45,48^. Most of the studies employed more than one intervention (Table 2).

**Table 2.** Description of the interventions carried out

Interventions	Apisarnthanarak, 2015	Abubakar, 2019	Bergh, 2020	Cheng, 2023	Díaz-Madriz, 2020	Du, 2020	Gebretekle, 2020	Holguín, 2020	Holguín, 2021	Jantarathaneewat, 2022	Kerr, 2021	Kuruvilla, 2023	Li, 2017	Mahmoudi, 2020	Masri, 2022	Messina, 2015	Nampoothiri, 2021	Wang, 2019	Wang, 2022	Xu, 2022	Yu, 2023	Zhang, 2019	Zhang, 2020	Zhou, 2021
Administration hang-time																								
Antibiotic prophylaxis																								
Audit and feedback																								
Consultation of treatment suggests																								
Culture analysis (de-escalation/escalation)																								
Daily rounds																								
Evaluation of antibiotic therapy (dosage, route, frequency)																								
Implementation of protocols infectious diseases																								
Indication evaluation																								
Patient counseling and education																								
Pharmacists training																								
Physician training																								
Reassessment of allergy history																								
Treatment duration assessment																								

Most of the outcomes reported involved reduction in antimicrobial consumption (12 studies)^25–30,37,38,41–44^, cost reduction (6 studies)^26,31,38,43,44,48^, reduction in the spectrum of action of antibiotics (6 studies)^32,33,37,39,45,48^, increased adherence to protocols (3 studies)^34,35,40^, and reduction in hospitalization days (3 studies)^25,31,38^. Of the twenty-four studies included, only seven of them evaluated mortality as an outcome from which two observed a significant reduction.^31,37^ Out of all the studies, only three studies identified reduction in AMR^31,37,45^.

Regarding the quality assessment scores of the 24 included studies, five of them were considered good (19–23 of 28 possible points) per the Downs and Black quality tool^27,32,44,46,47^. More than half of the studies (17 studies) were considered fair (14–18 points)^25,26,28–31,33,34,36–40,42,43,45,48^, and two studies were considered poor quality (≤13 points)^35,41^.

## Discussion

In our systematic review, we found that ASPs are associated with decreased antibiotic utilization in multiple studies conducted in LMICs. Between a variety of different ASPs tasks and interventions, the main ones were audit and feedback, education, training, implementation of protocols, and daily rounds, which were mostly led by non-ID-trained pharmacists. Previous systematic literature reviews have also shown that pharmacist-led ASP improved the correct antibiotic prescription and adherence to guidelines in countries with high rates of inappropriate prescription.^49–51^ However, none of them focused only on LMICs. In our systematic review, we observed that clinical pharmacists are key professionals well prepared to manage these programs and are successfully involved in implementing ASPs interventions in LMIC inpatient settings. They play an important role by following protocols and contribute positively to the outcomes of these programs.

In most studies, the interventions were conducted by clinical pharmacists who were not specialized in IDs, and the outcomes obtained in their interventions were favorable, contributing to the reduction in the use of antimicrobials. For example, one study demonstrated the positive impact of a multifaceted clinical pharmacist-led program on antimicrobial stewardship through segmented regression analysis. A reduction in the quantity of antibiotic use after the ASPs intervention was seen, additionally, there was a significant decrease in the proportion of patients receiving more than one antibiotics and the average length of hospital stay.^30^ Through the review, we identified that more than half of programs did not have ID specialization, this can be justified by the lack of specific specializations degrees available in these countries. There is no formalization and obligation from hospitals to have an ID specialized pharmacist to manage the ASPs. In addition to this, other factors that prevents the progress of ASPs in some regions are the lack of adequate resources, technology and laboratory support, workforce, and support from leadership.^17,43,44^

In developing countries, such as Brazil, clinical pharmacists are trained in a wide range of activities and there are no postgraduate options in ID as in the United States. In most of the included studies on LMICs, management programs were led by pharmacists without specialization in ID. The ID training consists of training focused on antimicrobial therapy combining optimization strategies and broad knowledge in pharmacokinetics, pharmacodynamics, microbiology, and IDs. However, most of these pharmacists received basic training in antimicrobial management by the hospital infection control service’s medical team, qualifying them to lead different interventions. Despite the limited availability of ID-trained pharmacists as well as ID physicians in some of these settings, clinical pharmacists already obtain successful outcomes in reducing AMR, encouraging further investments in education and training.

In the United States, ASPs allows multiple pharmacist-led activities, led by both clinical pharmacists and ID pharmacists. This review shows that antimicrobial stewardship interventions led by clinical pharmacists are an alternative solution found by LMICs. Several studies evaluated the effectiveness of clinical pharmacists’ interventions, described the development of ASPs, and elucidated the role of pharmacists in these programs. The outcomes and benefits achieved by these practices were determined and, consequently, multiple pharmacist-led interventions and protocols were identified.^18,50–55^

We found an increase in publications on ASPs in recent years from LMICs, mainly in countries classified as upper middle income, such as China, South Africa, and some countries in Latin America, demonstrating the importance of the contribution of pharmacists in the rational use of antimicrobials and in the fight against bacterial resistance. There were differences observed between the studies. In China, ASPs are more widespread as it is a country with more resources, receiving greater support from local authorities, so much so that the majority of the reviewed studies are from this country. They mainly involved auditing actions, feedback, and daily rounds. In Africa, we noticed that there is an incentive through partnerships with developed countries, helping to disseminate the importance of ASPs by providing support through training and implementation of protocols.^35^ In Latin America, we noticed mainly educational activities with two Colombian studies having the evaluation of antimicrobial prescriptions carried out by ID specialized pharmacists^32,33^, demonstrating that they receive support from the management team of these institutions.

In most of the studies there was either an overall reduction in the use of antimicrobials or in the use of a specific type of antibiotic, depending on the pharmaceutical intervention carried out. Moreover, as a consequence of the several interventions carried out by pharmacists, such as shorter treatment duration, discontinuation of unnecessary antibiotics, de-escalation from broad-spectrum to lower-spectrum drugs, switch therapy, and reduced duration of hospitalization, there was an overall cost reduction. Other outcomes, such as the decrease in mortality rate and in the microbial resistance rate were also observed in some of the studies but not all were able to achieve statistical significance. Although it is suggested that ASPs can prevent the increase in bacterial resistance rates, studies with longer intervention periods are needed to demonstrate the real impact.

Regardless of the country and the regional differences observed, different interventions were performed simultaneously to achieve positive results. It is possible to verify that hospitals with well-structured ASPs in LMICs have a variety of interventions and protocols that can be implemented and successfully managed by pharmacists.^37,43,48^ It is worth mentioning that in recent years, some countries have increased the participation of clinical pharmacists in ASPs^12^ and this increase is likely underestimated. Education, training, and protocol development are, in short, the components for initial implementation^49,56,57^ These interventions combined with auditing and feedback are present in most of the studies analyzed in this review. The latter being necessary to compose strategies and lead to better outcomes in the ASPs.

Our study had some limitations. First, the search was carried out only in one database. Second, no meta-analysis was carried out. Third, most studies were classified as having poor quality by the Downs and Black quality tool. Lastly, there was great heterogeneity between studies, such as different populations, great variation between study periods, and no description of which antimicrobials were evaluated, and how interventions were carried out. Despite the importance of the pharmacist and the positive impact on patient outcomes, there is still a lack of studies with strong evidence to corroborate this practice. Therefore, our review suggests that further studies should be conducted in this regard, mainly in LMICs.

In LMICs, we would expect that most pharmacists would not have expertise in ID. Nonetheless, 66% of studies within our review had pharmacists with comparable skill, which is a positive and promising number. It shows that, in recent years, hospital management has supported this cause by implementing ASPs and focusing on training and education of pharmacist in ID; therefore, contributing to the rational use and reduction of AMR. In our perception, ASPs must have at least one ID pharmacist who disseminates their knowledge by conducting trainings focused on strategic interventions led by clinical pharmacists.

Our findings reinforce the importance of clinical pharmacists´ participation in this context and the importance of a global need to increase ID training to continue to expand the impact of these healthcare professionals on ASPs, especially in LMICs.
